# Human Retrotransposons and the Global Shutdown of Homeostatic Innate Immunity by Oncolytic Parvovirus H-1PV in Pancreatic Cancer

**DOI:** 10.3390/v13061019

**Published:** 2021-05-28

**Authors:** Matthias Neulinger-Muñoz, Dominik Schaack, Svetlana P. Grekova, Andrea S. Bauer, Thomas Giese, Gabriel A. Salg, Elisa Espinet, Barbara Leuchs, Anette Heller, Jürg P. F. Nüesch, Miriam Schenk, Michael Volkmar, Nathalia A. Giese

**Affiliations:** 1 Department of Surgery, European Pancreas Center, University Hospital Heidelberg, 69120 Heidelberg, Germany; neulingermatthias@gmail.com (M.N.-M.); grekova.svitlana@googlemail.com (S.P.G.); GabrielAlexander.Salg@med.uni-heidelberg.de (G.A.S.); anette.heller@gmx.net (A.H.); m.schenk@med.uni-heidelberg.de (M.S.); 2 Department of Anesthesiology, University Hospital Heidelberg, 69120 Heidelberg, Germany; Dominik.Schaack@med.uni-heidelberg.de; 3 German Cancer Research Center (DKFZ), Division of Functional Genome Analysis, 69120 Heidelberg, Germany; andrea.bauer@dkfz-heidelberg.de; 4 Institute of Immunology and German Center for Infection Research (DZIF), Partner Site Heidelberg, University Hospital Heidelberg, 69120 Heidelberg, Germany; Giese@uni-hd.de; 5 German Cancer Research Center (DKFZ), Division of Stem Cells and Cancer, 69120 Heidelberg, Germany; e.espinet@dkfz-heidelberg.de; 6 HI-STEM—Heidelberg Institute for Stem Cell Technology and Experimental Medicine GmbH, 69120 Heidelberg, Germany; 7 German Cancer Research Center (DKFZ), Division of Tumor Virology, 69120 Heidelberg, Germany; b.leuchs@dkfz-heidelberg.de; 8 German Cancer Research Center (DKFZ), Division of Virus-Associated Carcinogenesis F170, 69120 Heidelberg, Germany; jpf.nuesch@dkfz-heidelberg.de; 9 German Cancer Research Center (DKFZ), Division of Molecular Oncology of Gastrointestinal Tumors, 69120 Heidelberg, Germany; m.volkmar@dkfz-heidelberg.de

**Keywords:** oncolytic parvovirus H-1PV, innate immunity, interferon, ISG, HERV, pancreatic cancer

## Abstract

Although the oncolytic parvovirus H-1PV has entered clinical trials, predicting therapeutic success remains challenging. We investigated whether the antiviral state in tumor cells determines the parvoviral oncolytic efficacy. The interferon/interferon-stimulated genes (IFN/ISG)-circuit and its major configurator, human endogenous retroviruses (HERVs), were evaluated using qRT-PCR, ELISA, Western blot, and RNA-Seq techniques. In pancreatic cancer cell lines, H-1PV caused a late global shutdown of innate immunity, whereby the concomitant inhibition of HERVs and IFN/ISGs was co-regulatory rather than causative. The growth-inhibitory IC50 doses correlated with the power of suppression but not with absolute ISG levels. Moreover, H-1PV was not sensitive to exogenous IFN despite upregulated antiviral ISGs. Such resistance questioned the biological necessity of the oncotropic ISG-shutdown, which instead might represent a surrogate marker for personalized oncolytic efficacy. The disabled antiviral homeostasis may modify the activity of other viruses, as demonstrated by the reemergence of endogenous AluY-retrotransposons. This way of suppression may compromise the interferogenicity of drugs having gemcitabine-like mechanisms of action. This shortcoming in immunogenic cell death induction is however amendable by immune cells which release IFN in response to H-1PV.

## 1. Introduction

The oncolytic parvovirus H-1PV has already entered clinical trials targeting glioblastoma and pancreatic cancer [1,2], but ultimately curing protocols are yet to be generated. A promising way to achieve this goal is the induction of the immunogenic form of cell death (ICD), which efficiently couples oncolysis with immune activation triggering long-term anticancer responses [3]. Oncolytic parvoviruses engage the immune system to suppress the growth of tumors in addition to direct oncolytic effects and thus, promote host survival [4,5,6,7,8]. In pancreatic cancer (PDAC), the lack of complete tumor eradication might be due to the restricted ability of H-1PV to fully induce ICD. Out of the three major ICD determinants, H-1PV robustly triggered the production of high-mobility group box 1 (HMGB1) and, at least partially, ATP but not the translocation of calreticulin to the cell surface (ecto-CALR) [9]. Since the function of the latter was recently reassigned from an “eat-me” signal to type I interferon (IFN-I) triggering, the ecto-CALR-independent induction of IFN emerged as an alternative route to achieve ICD and promote the desired T-cell mediated anti-tumor immunity [10,11].

The single-stranded (+) parvoviral DNA contains only two major open reading frames encoding for two non-structural (NS1 and NS2) and two structural capsid proteins (VP1 and VP2), whose expression is controlled by alternative splicing. Additionally, their activity is modulated by post-translational modifications through cellular kinases [12,13,14]. These processes significantly diversify the functional spectrum of the viral effectors. The genomic simplicity, multifaceted interactions with host factors and cell cycle-dependency determine the parvoviral oncotropism [15,16]. Non-transformed cells usually undergo an abortive infection, whereby a low proliferative activity is not the sole restrictive factor. The activation of innate antiviral IFN-stimulated genes (ISGs) as well as the production of IFN and antibodies in immune cells, efficiently preclude the extra-tumoral distribution of a parvoviral infection, thus ensuring the non-pathogenic character of oncolytic parvoviruses in humans [1,17,18]. In tumor cells, clinically suitable autonomous parvoviruses (e.g., H-1PV, MVMp, and LuIII) do not seem to alter the IFN expression, at least early after infection [18,19,20,21]. Then, immune cell-derived IFN may represent a way to overcome the lack of ecto-CALR translocation in PDAC lesions, which are well-known for immune infiltration and cellular heterogeneity [22,23,24,25]. In this context, the sensitivity of H-1PV to IFN and antiviral effector ISGs in tumor cells becomes an important clinical issue. Pre-treatment with IFN did not affect early stage H-1PV infection in glioma, sarcoma, and melanoma cells [20]; later or PDAC responses are not studied yet.

Intriguingly, parvoviruses do not display a common oncotropic strategy toward IFN/ISGs. Overall, their net effect on IFN triggering and signaling seems to be inhibitory, if any. A plasmid encoding the full-length genome of parvovirus B19 (primate erythroparvovirus 1 causing erythema infectiosum in humans) was shown to not only trigger IFN production but also to suppress the JAK/STAT signaling pathway, thus blunting IFN-responsiveness in HeLa cells [26]. MVMp (Minute virus of mice, prototype strain) induced but did not succumb to IFN in murine embryonic fibroblasts while becoming unresponsive to poly-(I:C), a synthetic analog of double-stranded DNA to activate an antiviral response. There, MVMp also promoted the phosphorylation of eIF2α and activated PKR (protein kinase RNA-activated/EIF2AK2); this PKR-mediated antiviral response was impaired in human transformed cell lines [27,28]. FPV (Feline panleukopenia virus, a carnivore protoparvovirus 1 strain Philips-Roxane) did not activate IFN-I expression but antagonized the induction of an IFN response stimulated by Sendai virus in F81 cells. This antagonizing effect was mediated by the parvoviral NS2 protein, which disrupted an interaction between TANK-binding kinase (TBK1) and stimulator of interferon genes STING (TMEM173) and thus inhibited the phosphorylation of IRF3, the major components of the afferent IFN-I pathway [29]. Among other effects, this blockade disabled the cGAS (cyclic GMP-AMP synthase)/STING-mediated sensing of nucleic acids—an afferent interferogenic pathway also activated by gemcitabine, a standard genotoxic anti-PDAC drug with broad antiviral activity and the ability to trigger IFN-dependent immunity in dying tumor cells [30,31,32,33,34]. Characteristically, gemcitabine does not impede the production of parvoviral determinants (nucleic acids and proteins) in PDAC cells [9]. By antagonizing the IFN response in tumor cells, H-1PV might diminish the potential of gemcitabine to induce immunogenic cell death while synergizing with oncolytic activity.

PDAC cells are highly ISG-abundant per se (so-called “viral mimicry state”), mount an IFN response to viruses such as VSV, NDV, VVT7, HSV-1, or myxoma and respond well to exogenous IFN [35,36,37]. Despite entering clinical trials NCT01301430 and NCT02653313, one of the most basic features to determine H-1PV’s therapeutic efficacy—its interference with innate immunity—remains unclear. In 1993, Faisst et al. showed that the parvoviral NS1 protein sequesters transcription factor NF-κB from the long terminal repeat (LTR) of human immunodeficiency virus 1 (HIV-1) and thus inhibits HIV-promoter activity [38]. Recently, the LTR-containing autonomous retrotransposons (human endogenous retroviruses, i.e., HERVs) emerged as a major trigger for constitutive antiviral signaling in malignant cells, including PDAC, and also in certain normal cells [35,39,40,41,42,43,44]. HERVs configure the innate antiviral state by producing dsRNA that triggers a type I or III IFN-dependent ISG response to function as a HERV controller. Since NF-kB may drop upon H-1PV infection in PDAC cells [45], we hypothesized that parvoviral NS1/2 may cause a delayed inhibition of antiviral immunity by interfering with the expression of IFN-stimulatory HERVs. This may have far-reaching oncolytic and immunogenic implications, positive or negative. The current study attempted to reconstruct the interactions of H-1PV with HERVs, ISGs, and type I/III interferons in pancreatic cancer.

## 2. Materials and Methods

### 2.1. Cell Culture and Treatments

Peripheral blood mononuclear cells (PBMCs) were obtained from healthy donors using the standard density gradient separation method (Histopaque-1077; Merck KGaA, Darmstadt, Germany). Pancreatic tumor cells (PDACs) were represented by a panel of nine cell lines: commercially available AsPC1, BxPC3, Capan1, Colo357, MiaPaca2, Panc1, SU8686, and T3M4 and the freshly established ASAN-PaCa [46]. Authentication was done via STR-DNA-Typing as a service provided by the DSMZ (German Collection of Microorganisms and Cell Cultures GmbH, Braunschweig, Germany). Mycoplasma testing was performed by PCR. Cells were grown in RPMI-1640 medium supplemented with 10% fetal bovine serum, 100 U/mL penicillin, and 100 µg/mL streptomycin. Recombinant human interferon-alpha and interferon-beta were purchased from PBL Assay Science (Piscataway, NJ, USA) and used at a concentration of 1000–2000 U/mL. The treatment included continuous exposure for 72 h and pulse exposure for 8 h at different time points post H-1PV infection. 

### 2.2. H-1PV Production and Infection

Wild-type H-1PV, recombinant GFP-H-1PV, and empty capsids were produced as detailed previously [47,48,49]. In short, wild-type H-1PV was produced by infecting NBK cells and purifying released virions by using iodixanol gradient centrifugation and dialysis against Ringer solution. Virus titers were measured by standard plaque assays and expressed as numbers of PFU per mL. Virus stock contamination with endotoxin was less than 2.5 endotoxin units (EU)/mL. The pancreatic cancer cells were seeded at densities of 1.5–2.5 × 10^5^ cells/2 mL in 6-well plates, depending on the growth rate of each cell line, while PBMCs were seeded at a density of 1 × 10^6^ cells/mL. The next day, the cells were infected with wild-type H-1PV at a multiplicity of infection (MOI) of 10 PFU/cell. Empty capsids and GFP-H-1PV were added at an amount matching the absolute numbers of particles of wild-type H-1PV. GFP-H-1PV was produced infecting 293T cells using the PEI-method. Stock solutions of GFP-H-1PV were kept at a concentration of 1.7 × 10^10^ genome-containing particles/mL and empty capsids at 4.9 × 10^13^ physical particles/mL. Cells and supernatants were collected and assayed at 0–72 h post-infection (hpi). The growth-inhibitory IC50 dose (concentration which reduces the growth by 50%) of each cell line was determined by infecting them using the dilution rows of H-1PV from 0 to 100 MOI as PFU/cell. The cell viability was assessed 72 hpi by crystal violet staining. After wash steps to remove the detached cells, the quantification of viable cells was achieved by dissolving the incorporated dye with methanol and measuring the optical density at 595 nm.

### 2.3. Transfection-Based Experiments

The human ERVWE-1 (NM_014590.3) bearing vector pCMV6-XL4-Vector (AF067196) and control plasmid were obtained from Origene (Rockville, MD, USA). The transfections were performed on three pancreatic cancer cell lines (AsPC1, MiaPaca2 and T3M4) in a 6-well plate format using the TurboFect reagent according to the manufacturer’s instructions (Thermo Fisher Scientific Inc., Waltham, MA, USA). siRNA-based knockdown was performed with Lipofectamine RNAimax reagent (Thermo Fisher Scientific Inc.) to transfect commercially available esiRNA sets (10 nM) targeting ERVWE1 or negative control EGFP esiRNA (Merck). Cells and supernatants were harvested and analyzed at 24–96 hpi.

### 2.4. Western Blot Analysis

The protein expression of ISG15, IFITM1 and beta-actin in RIPA-lysed cells was analyzed by NuPage-based Western blotting and ECL-based detection as previously described [9]. Visualized band intensities were quantified using ImageJ software, normalized to β-actin values, and expressed as a percentage of the respective control expression level.

### 2.5. ELISA

Measuring type I and III interferons in the medium conditioned by pancreatic tumor cells was performed using commercially available kits from PBL Assay Science and Invitrogen by Thermo Fischer Scientific Inc.

### 2.6. Real-Time qRT-PCR

The expression of viral (NS1) and cellular (ISGs and HERVs) transcripts was analyzed by qRT-PCR detailed in previous publications [4,9,50]. The commercially available MagNAPure LC HS mRNA isolation kit and cDNA synthesis kit were provided by RAS (Mannheim, Germany). PCR reagents and primers for qPCR suitable for a LightCycler480 were provided by Search-LC (Heidelberg, Germany). Normalization was performed by relating the number of detected transcripts to 10 k copies of the housekeeping gene PPIB. For amplicons not spanning introns, e.g., HERV-W1env, we additionally controlled results by conducting qPCR using DNAse-treated RNA preparation as a template (“minus reverse transcriptase” control). Additional confirmation of HERV expression was performed by conventional PCR using published primer sequences and visualizing PCR products using ethidium bromide-stained agarose gels.

To estimate the impact of H-1PV on the expression of marker ISGs (IFITM1, ISG15 and OAS1) and HERVs (HERV-Kenv, HERV-Wpol, HERV-Wenv) in PDAC and immune cells, we calculated the ratio between the transcripts detected in infected and non-infected cells for each marker gene and Log2-transformed values (fold-change calculation). The mean expression of three marker genes served as a single “ISGs” or “HERVs” parameter. Each cell line was repeatedly tested two to nine times to yield the mean ISGs and HERVs values per cell line. These numbers have been used to calculate “PDAC” mean ± SEM (*n* = 9 for nine cell lines per group; *n* = 3 for three cell lines per group).

### 2.7. RNA Sequencing

Transcriptome profiling was carried out by the core facility of DKFZ (H-1PV-treated cells) and EMBL (constitutive expression in nine PDAC cell lines), both located in Heidelberg, Germany. Total RNA was isolated from the cell cultures using the RNeasy Mini kit (Qiagen, Hilden, Germany) according to the manufacturer’s instructions. After isolation, the total RNA was treated with the Turbo DNA-free kit according to the manufacturer’s instructions (Thermo Fisher Scientific). The RNA concentration and quality were evaluated by Nanodrop and Agilent2000 Bioanalyzer. RNAseq libraries were prepared using the TruSeq stranded total RNA kit and sequenced using an Illumina HiSeq 4000 platform, producing 50 bp single-end reads ranging from 36 to 72 million reads per sample. Quality control of the RNAseq FastQ files was performed with FastQC v.0.11.8 [51]. The obtained reads were pseudoaligned using the hg38 reference genome and quantified by Salmon v1.2 with standard parameters. The resulting transcript expression levels were summarized to gene-level expression values and corrected for average transcript length by using tximport v1.10.1 [52] and the “lengthScaledTPM” option while filtering out low expressed genes (average counts < 10). Differentially expressed genes for the pancreatic cancer cell lines were determined by using the DESeq2 v1.22.2 package [53]. Using the DESeq2 and log2-fold change of pre-ranked differentially expressed genes, a gene set enrichment analysis was performed using the fgsea package v1.8 and the hallmark gene sets from MSigDB v7.1 [54]. Furthermore, transcripts per million (TPM) expression levels of endogenous retroviruses and repetitive elements were estimated using SalmonTE pipeline v0.4 [55], which utilizes a Repbase-derived reference genome (https://www.girinst.org/, accessed on 27 April 2020) [56]. The GEO accession numbers are GSE160434 and GSE160322.

### 2.8. Statistical Analyses

As a rule, measured values were averaged for each cell line and experiment before calculating average “PDAC” values at each time point. The difference between two conditions was calculated as a fold change: log 2-transformed ratio of treatment or infection to control (Log2FC). The figures show these data as mean values ± the standard error of the mean (SEM). Statistical significance was tested by performing a two-sided t-test or Mann–Whitney test, (upon testing for a normal distribution). The correlation of the data between different groups was assessed using Pearson’s test. The level of significance was set at *p*-value < 0.05. Data analysis and presentation were done with GraphPad Prism software and R version 3.5.2 with additional packages tidyverse v1.3, ggpubr v0.4, and ggrepel v0.8.2 (https://www.R-project.org/, accessed on 4 May 2021). The heatmaps were generated using the packages ComplexHeatmap v2.5.4 and circlize v0.4.10 [57,58].

## 3. Results

### 3.1. Concomitant Suppression of ISGs and HERVs in H-1PV-Infected PDAC Cells

**Figure 1:**
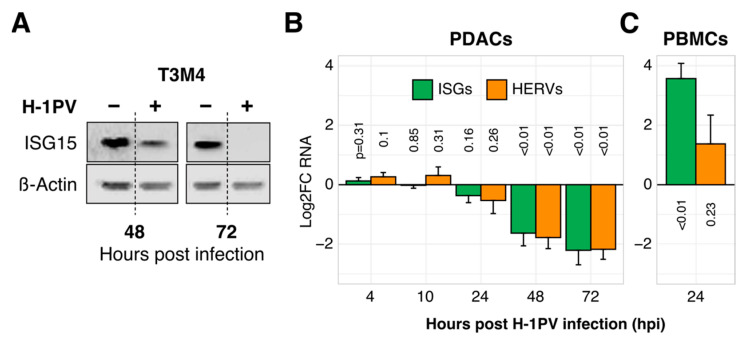
The oncolytic parvovirus H-1PV suppresses ISG and HERV expression in tumor but not in immune cells. (**A**) Illustrative reduction of ISG15 protein expression upon H-1PV infection in the pancreatic cancer cell line T3M4 (western blot analysis). (**B**) Suppression of ISG and HERV RNA expression in PDAC cells according to the Log2-transformed infection-to-control ratios (Log2-fold change, i.e., Log2FC). Expression of marker ISG (ISG15, IFITM1, OAS1) or HERV (HERV-Kenv, HERV-Wpol, HERV-W1env) genes was measured by qRT-PCR in pancreatic cancer cell lines (PDAC, *n* = 9) with or without exposure to H-1PV at MOI = 10 PFU/cell. Each kinetic was repeated two to nine times per cell line before calculating the mean ± SEM for nine cell lines. (**C**) Induction of ISG and HERV expression by H-1PV 24 hpi in immune cells (peripheral blood mononuclear cells, i.e., PBMCs, *n* = 5). Abbreviations: ISG, interferon (IFN)-stimulated genes; HERV, Human Endogenous Retroviruses; PDAC: pancreatic cancer; MOI, multiplicity of infection; PFU, plaque-forming units.

To explore the relationship between H-1PV and innate immunity in pancreatic cancer, we infected nine PDAC cell lines and monitored the expression of three marker ISGs (ISG15, IFITM1, OAS1) and three marker HERVs known for establishing an antiviral state in tumor cells (HERV-K env, HERV-W env, HERV-W pol) [43]. As shown in Figure 1A,B, ISG as well as HERV levels dropped profoundly during the infection of PDAC cell lines, both at an RNA and protein level. This previously unknown parvoviral feature set in by 30 hpi and progressed thereafter. In contrast, immune cells expectedly upregulated ISGs together with a modest accumulation of HERVs in response to H-1PV by 24 hpi (peripheral blood mononuclear cells/PBMCs; Figure 1C).

Most probably, different onset and direction of antiviral reactivity between immune and tumor cells are determined by the ability to sense early parvoviral components such as newly synthesized nucleic acids. Immune cells mount an early TLR9/IFN-dependent response to abort the infection prior to the synthesis of viral proteins [18]. Following this reasoning, tumor cells should lack this early recognition and thus allow H-1PV’s replication to proceed toward the production of parvoviral proteins. Upon accumulation, some of these proteins will suppress the expression of ISGs and HERVs. To test this assumption, we infected three PDAC cell lines (AsPC1, MiaPaca2, and T3M4) with the recombinant GFP-H-1PV or with empty viral particles (Figure S1). The former contains DNA encoding NS but not VP proteins, the latter lack the entire genome. GFP-H-1PV was able to inhibit ISG expression (Log2FC = −0.52 + 0.24, *p* < 0.05), whereas empty capsids did not cause any change in ISG expression (Log2FC = +0.05 ± 0.18, *p* > 0.05). Thus, the non-structural NS1/NS2 proteins are essential for ISG suppression but capsid VP1/2 proteins are not, either newly synthesized or assembled.

### 3.2. Suppressed Type I and III IFNs Are Co-Targets Rather Than Regulators of ISGs

HERVs are believed to engage the innate immunity in tumor cells via IFN-I (IFNα and IFNβ) or IFN-III (epithelial IFNλ i.e., IL28 and IL29) [35,42,43,44]. An analysis of supernatants taken from both infected and non-infected PDAC cultures revealed the absence of secreted IFN-I proteins (ELISA and bioassay, a courtesy measurement by R. Zawatzky at DKFZ, Heidelberg). Analysis of the cellular RNA content by qRT-PCR showed limited accumulation of IFN-I transcripts during prolonged culture in two out of nine tested cell lines (ASAN-PaCa and T3M4; up to 10 transcripts/10kPPIB) (Figure S2A). Similar to ISGs and HERVs, this expression was eliminated by H-1PV. In contrast to IFN-I, an expression of IL29 was a common PDAC event, with an increasing accumulation of IFNλ (IL29) protein in the supernatants of all nine tested cell lines up to 100 ng/mL within three days of culture. Parvoviral infection ubiquitously reduced only this “late” level by 20 ± 7%, but not early levels (Figure S2B). Thus, IFN-I and IFN-III represent unlikely candidates to account for ISG reduction under H-1PV in PDAC cells in general or to link HERVs and ISGs in particular.

### 3.3. Restricted Ability of HERVs to Regulate ISG Expression

**Figure 2:**
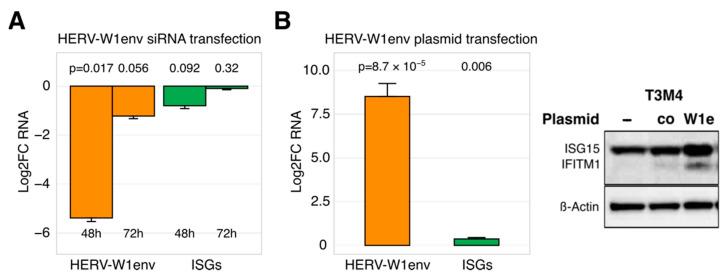
HERVs may control the expression of ISGs in PDAC cells. (**A**) Almost complete siRNA-based elimination of HERV-W1env in T3M4 cells tends to reduce ISG levels within 48 h. The knockdown effect is however not persistent and rapidly diminishes, together with the ISG-effect (*n* = 3). (**B**) Drastic overexpression of HERV-W1env by plasmid-based transfection in PDAC cell lines (AsPC1, MiaPaca2, T3M4) caused a modest increase in ISG as measured by qRT-PCR and Western blot analyses. Each experiment was repeated twice. Abbreviations: ISG, interferon (IFN)-stimulated genes; HERV, Human Endogenous Retroviruses; PDAC: pancreatic cancer.

To address the question whether HERVs may engage innate immunity in PDAC cells at all, we used transfection-based approaches to modify HERV levels and performed qRT-PCR and Western blot to measure the ISG outcome. As shown in Figure 2A,B, artificially dropping or raising HERV RNA content respectively decreased or increased ISG expression. It should be noted that the regulatory effect was conditional: only a drastic change in expression of a single HERV entity caused an at least measurable impact on ISGs. Out of three HERV-targeting siRNA sets, solely the complete knockdown of HERV-W1env in T3M4 cells (<1% RNA remaining at 48 h post-transfection) tended to reduce IFN-I and ISG levels by 50% (*p* = 0.09; Figure 2A); IFN-III (IL29) expression was not changed. This inhibitory effect vanished with rapid restoration of HERV RNA content (~50% at 72 h post-transfection, Figure 2A) and did not affect the protein abundance of ISGs. In three tested PDAC cell lines, artificial overexpression of HERV-W1env with the gene-bearing plasmid had to be increased 10.0-fold to achieve a 0.5-fold elevation in ISGs and a modest protein gain (Figure 2B). In principle, our findings corroborated the ability of selected HERVs to impact the antiviral state in PDAC cells. The expression levels necessary for the control function, however, laid out of the fluctuation range observed among the tested cell lines. Furthermore, the constitutively expressed HERVs, IFNs, and ISGs did not correlate with each other. In PDAC cells, H-1PV seems to disturb the innate immunity by targeting host factors capable of regulating HERVs, IFNs, and ISGs simultaneously, i.e., in a co-regulatory, non-hierarchical manner.

### 3.4. Global Disturbance of Innate Immunity by H-1PV Associates with a Clade-Specific Distortion of Retrotransposons

**Figure 3:**
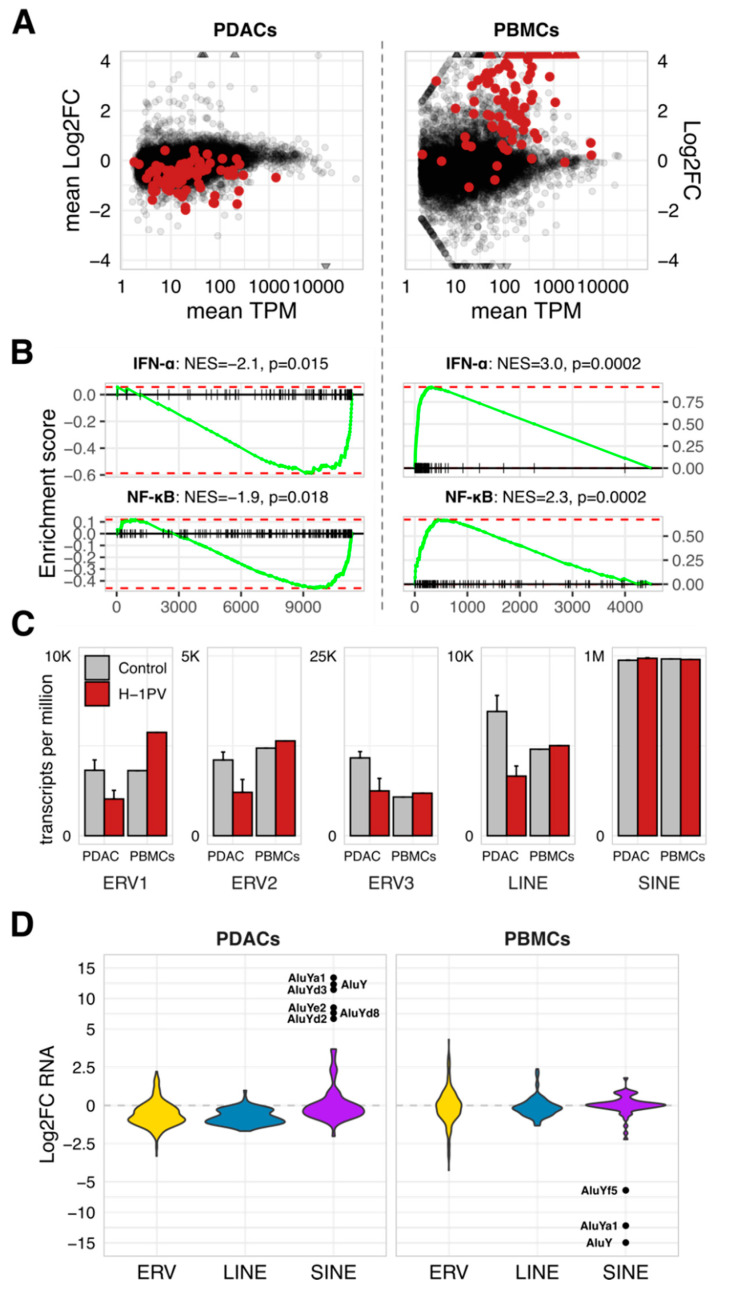
The global shutdown of innate immunity in tumor cells is associated with clade-biased deregulation of endogenous retrotransposons. RNAseq-based comparison of H-1PV-infected vs. non-infected cells revealed global suppression of antiviral innate immunity in the PDAC cells (AsPC1, MiaPaca2, and T3M4 taken at 48 hpi) and global stimulation—in the normal immune cells (PBMCs taken at 24 hpi). (**A**) Distribution of the transcripts according to mean TPM (transcripts per million) level in non-infected cells versus mean Log2FC values. Red dots denote gene entities defining the Hallmark IFN-alpha signature. (**B**) GSEA output with the pathways significantly altered by H-1PV in infected cells. (**C**) Clade-specific deregulation of HERV expression is shown using TPM values to visualize abundance and differences among major classes in the control and infected PDACs and PBMCs. The data are given as means per group. (**D**) RNAseq-detected expression of the endogenous retrotransposons as quantified using the SalmonTE/RepBase-pipeline and split into the ERV1-3, LINE, and SINE clades for the graphic presentation of the mean Log2FC values. Apparent IFN/ISG-sensitive outliers in the SINE-clade are labeled to indicate belonging to the AluY-cluster. Abbreviations: ISG, interferon (IFN)-stimulated genes; HERV, Human Endogenous Retroviruses; PDAC: pancreatic cancer; PBMCs, peripheral blood mononuclear cells; GSEA, Gene Set Enrichment Analysis; LINE and SINE, long- and short interspersed nuclear elements.

To estimate the extent of an antiviral disturbance, we performed genome-wide profiling in infected and non-infected PDACs (pancreatic tumor cell lines AsPC1, MiaPaca2, and T3M4 at 48 hpi) and PBMCs (normal immune cells at 24 hpi). The RNA expression of human genes and retrotransposons was measured using RNAseq and quantified using the Salmon and Salmon-TE pipelines. This approach exposed the broad impact of H-1PV on gene expression (Figure 3A). Nevertheless, only alterations in IFNα and TNF/NF-κB responses separated infected PDACs and PBMCs (*p* < 0.05; among 50 Hallmark pathways maintained in the Molecular Signatures Database (MSigDB), Gene Set Enrichment Analysis; https://www.gsea-msigdb.org/gsea/msigdb, accessed on 14 August 2020; Figure 3B). H-1PV infection globally distorted innate immunity, from danger/pathogen sensors to signal transducers to end effectors. Notably, the inhibitory strength differed among the tested PDAC cell lines (Figure S3).

Retrotransposon-specific RNA occupied up to 0.4% of the studied transcriptomes, with 98% of these transcripts being non-autonomous short-interspersed nuclear elements (SINEs, Figure 3C). H-1PV infection does not alter SINEs expression, except for a small AluY-cluster (Figure 3D). That subset was upregulated in PDACs and downregulated in PBMCs, thus opposing the concomitant IFN/ISG changes—in agreement with Williams et al. reporting increased B1 and B2 SINE levels upon productive infection of the A9 cells by MVMp [59]. In contrast, autonomous HERV and LINE-sequences (long-interspersed nuclear elements) experienced ISG-like shutdown in parvovirus-infected PDACs. Infected PBMCs elevated only the ERV1 family (Figure 3C). Non-autonomous (SINEs) and autonomous (HERV, LINE) clades thus differ in their sensitivity to both, upstream regulation and ISG status.

Together with the transfection-based data, such clade specificity implied that in PDAC cells, H-1PV has a two-fold effect on retrotransposons. Primarily, it blocks a central homeostatic hub co-regulating IFNs/ISGs and HERVs/LINEs, some of which may directly control ISGs without succumbing to them. Apparently, the SINE-retrotransposons are not a part of this module (Figure 3D). Nevertheless, some SINEs—AluY-elements—are IFN-sensitive. Their reemergence in parvovirus-infected tumor cells is thus a secondary effect, i.e., a consequence of the ISG shutdown and a proof of its functionality. In addition to the marker function, the re-expression of AluY-elements in tumor cells may also have pathological and/or therapeutic consequences [60].

### 3.5. H-1PV Does Not Succumb to IFN but Blunts Its Triggering by Gemcitabine

**Figure 4:**
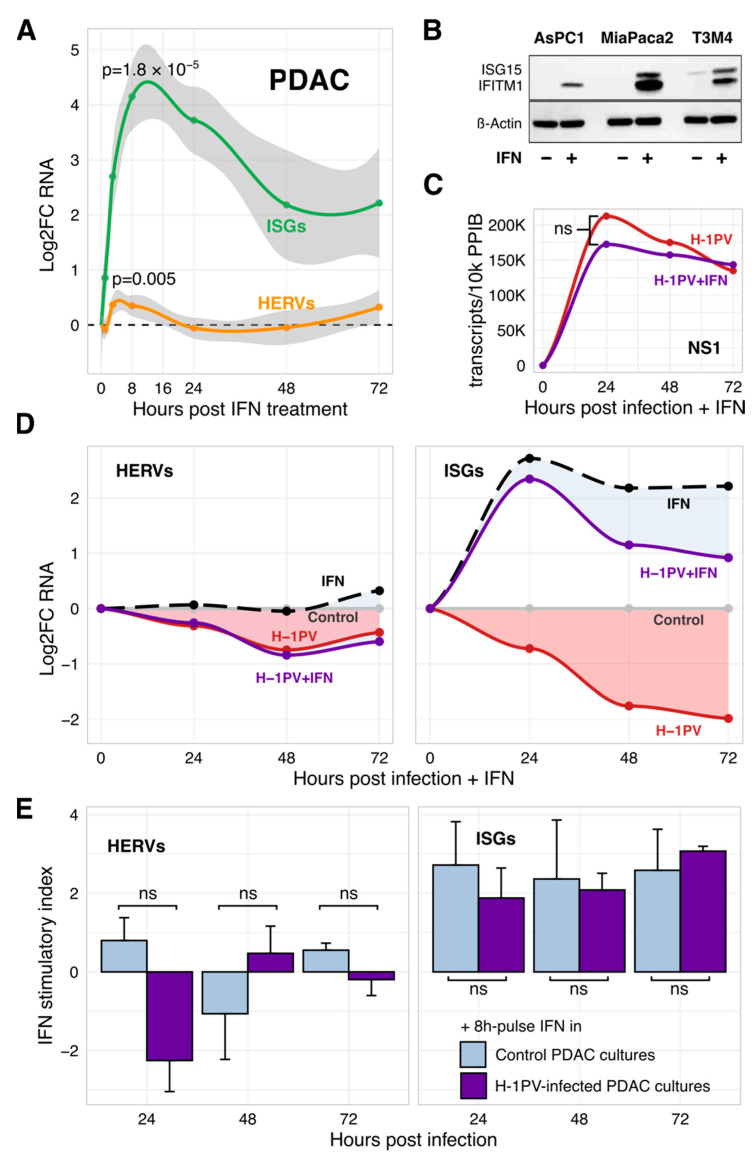
H-1PV is uncoupled from efferent IFN signaling in PDAC cells. (**A**) In nine tested PDAC cell lines, IFN upregulated expression of both ISGs and HERVs. The effect peaked at 8 h of treatment with 1000–2000 U/mL of IFNα or IFNβ (IFN-I) and declined thereafter but to a different extent (qRT-PCR). The curves represent mean Log2FC ± SEM values for treated vs. control conditions. Each cell line was tested two to five times; IFNα and IFNβ data were combined. (**B**) Continuous activation of ISGs by IFN-I (Western blot) did not suppress HERVs below basal levels. (**C**) Lack of significant difference (ns = non-significant; *p* ≥ 0.05) in the amount of the parvoviral NS1 transcripts in H-1PV-infected PDAC cells with or without exposure to IFNα. (**D**) H-1PV does not impede responsiveness of the PDAC cells to exogenous IFN-I while maintaining homeostatic shutdown of HERVs and ISGs. PDAC cell lines AsPC1, MiaPaca2, and T3M4 were infected with H-1PV and continuously exposed to IFNα at 2000 U/mL. RNA expression of marker ISG and HERV genes was measured by qRT-PCR. Data points present an average of three mean values, calculated for each cell line tested independently two to three times and normalized to the virus- and/or IFN-free controls at each time point. Filled areas illustrate the power of the response to IFN in infected vs. non-infected cells. (**E**) as in (**D**) except that exposure to IFN was performed as an 8 h-long pulse treatment prior to collection of the control and H-1PV-infected PDAC cells at 24, 48, and 72 hpi. The bars represent the mean ± SEM of indices calculated as a ratio between RNA expression values measured in IFN-treated and non-treated cells. Blue and purple bars show triggering of the HERVs/IFNs in control and H-1PV-infected cultures, respectively. Abbreviations: ISG, interferon (IFN)-stimulated genes; HERV, Human Endogenous Retroviruses; PDAC: pancreatic cancer.

Our data clearly showed that the productive infection of tumor cells with H-1PV is associated with a profound inhibition of innate immunity. The question is why is it needed? PDAC cells are known to maintain the IFN-transducing machinery and activate ISGs in response to IFN or certain infections [35,36,37]. Our PDAC cell lines also abundantly expressed IFN-I receptors IFNRA1 and IFNRA2, as measured by qRT-PCR, FACS, and immunofluorescent staining; this pattern was not altered by H-1PV (Figure S4). Treatment of non-infected PDAC cells with 2000 U/mL recombinant IFNα or IFNβ caused a strong sharp Log2FC = +4.0-increase in ISG expression and a modest Log2FC = +0.4-elevation in HERV transcripts (Figure 4A). Peaking at 8 h, the IFN-triggered ISG and HERV expression values were interrelated (R = 0.76, *p* = 0.028, *n* = 9 cell lines). Expression of HERVs rapidly normalized thereafter without further decline below the basal levels (Figure 4A) despite continuing activation of antiviral effector ISGs (Figure 4B).

Suppression of homeostatic and/or IFN-activated ISGs thus might be helpful for a productive infection. Continuous exposure of H-1PV-infected PDAC cells to IFN-I however did not alter the expression of parvoviral RNA or oncolytic activity (Figure 4C and Figure S5). In turn, H-1PV did not abolish the ISG-stimulatory activity of IFN. Although the absolute ISG expression values in co-treated PDAC cells were lower than in “IFN-only” controls, this deficit was comparable to a basal loss caused by H-1PV under homeostatic conditions (Figure 4D). Confirmative, a pulse treatment of H-1PV-infected PDAC cells with IFN-I for 8 h at 24, 48, and 72 hpi upregulated the expression of ISGs at any stage of infection with the similar power as in non-infected cells; the effect on HERVs was mostly negligible (Figure 4E). First of all, this pattern separates the homeostatic IFN/ISG/HERV-regulatory pathway targeted by H-1PV from the canonical IFN-mediated response. Second, it questions the biological necessity of the global ISG knockdown.

Even if irrelevant for H-1PV itself, the IFN/ISG shutdown bears clinical consequences. On the one side, suppression of ISGs might explain the improved cytotoxicity of the standard anti-PDAC drug gemcitabine during complimentary application [9,61]. On the other side, H-1PV blocks the production of IFN which is crucial for the induction of immunogenic cell death and long-lasting anti-tumor immunity. Gemcitabine was recently attributed with IFN-inducing and anti-viral properties [32,34,62,63]. In nine tested PDAC cell lines, gemcitabine did not trigger IFN-I RNA expression de novo. However, it increased the IFN-I level by Log2FC = +1.3 (to an average level of 36 ± 14 transcripts/10k PPIB) in ASAN-PaCa and T3M4, the only two cell lines which accumulated IFNα/β transcripts constitutively, and lost them upon H-1PV infection (Figure S5A). H-1PV reduced the gemcitabine-triggered expression of IFN-I by 80%. Therefore, the failure of gemcitabine and H-1PV to completely eradicate pancreatic tumors despite synergistic oncolysis [4,9] might be attributed to a blunted interferogenicity. More general, the therapeutic induction of an immunogenic form of tumor cell death will not be achieved by a combination of H-1PV with gemcitabine-like drugs which employ cGAS/STING/TBK1-mediated signaling to trigger IFN production.

In summary, H-1PV does not surrender to or compromise the action of exogenous IFN-I while maintaining a concomitant IFN/ISG/HERV-shutdown. The exact mechanism of the homeostatic inhibition remains to be determined. Its specificity, however, has far-reaching clinical consequences: even if potentially irrelevant for parvoviral replication, it sensitizes tumor cells to other chemotherapeutics or other oncolytic viruses but impedes the interferogenicity of other drugs.

### 3.6. Oncolytic Efficacy of H-1PV in PDAC Cells Correlates with the Degree of ISGs Suppression but Not with Their Absolute Levels

**Figure 5:**
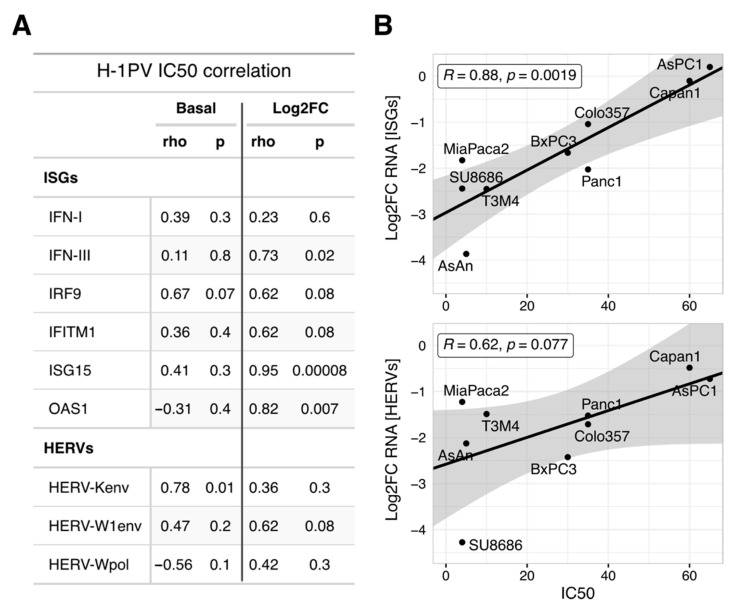
Oncolytic power of H-1PV in the PDAC cells correlates with the degree of the ISGs inhibition but not with their absolute levels. (**A**) Pearson rho-coefficients and *p*-values were calculated to estimate the correlation between H-1PV oncolytic activity (IC50 dose, PFU/cell) and expression of the marker IFN, ISG, or HERV genes taken as basal values or Log2-transformed infection-to-control ratios (*n* = 9 PDAC cell lines). (**B**) Pearson correlation between individual IC50 values and degree of downregulation for ISGs and HERVs calculated as a mean for three respective marker genes. A stronger capacity to suppress HERV/ISG at fixed MOI = 10 PFU/cell (i.e., most negative Log2FC value) indicates better oncolytic efficacy (i.e., lowest IC50 dose). A linear regression model was used to visualize the trend, the shaded area represents the standard error. Abbreviations: ISG, interferon (IFN)-stimulated genes; HERV, Human Endogenous Retroviruses; PDAC: pancreatic cancer; IC50, inhibitory concentration which reduces the cell growth by 50%; MOI, multiplicity of infection; PFU, plaque-forming units.

Most likely, the suppression of a hypothetical homeostatic HERV/IFN/ISG-module is an obligatory byproduct of a productive infection. In other words, this transcriptional module is accidentally controlled by a host protein whose disturbance is required for parvoviral replication and/or oncolysis. This would predict that the oncolytic potential of H-1PV will correlate with the ability to suppress ISGs, but not with their absolute levels. The oncolytic activity of H-1PV is known to differ among PDAC cells, with growth inhibitory IC50 doses ranging from 5 to 60 PFU/cell [4,9]. Indeed, the IC50 levels did not correlate with absolute levels of ISG and HERV expression, basal or parvovirus modified (Pearson’s r coefficients ranging from −0.45 to 0.74; *p* > 0.05 and Figure 5A). In contrast, the oncolytic efficacy strongly correlated with the degree of ISG downregulation (Log2FC, Figure 5B upper panel). PDAC cell lines showing a high degree of HERV/ISG inhibition upon H-1PV infection were more sensitive to H-1PVs cytotoxic effects (Figure 5A,B). This result was not confounded by the absolute number or ratio of infected cells [9].

To conclude, the antiviral state of PDAC cells—homeostatic or IFN-primed—does not determine the oncolytic efficacy of H-1PV per se. The potential regulator of homeostatic IFN/ISG/HERV expression seems to have a broad functional spectrum. While being crucial for the parvoviral life cycle, this host factor is targeted by H-1PV; that interference concomitantly disturbs a number of the non-crucial processes, including homeostatic innate immunity. The identity of the regulator remains to be determined. In addition to the previously suspected NF-kB, we have several potential candidates, whereby recent publications and a preliminary GSEA-based comparison of the sensitive and resistant PDAC cell lines strongly suggest focusing on the E2F-DNMT1 axis (see Section 4 Discussion). Nevertheless, the “linked byproduct” status indicates that the depth of ISG/HERV shutdown reflects the degree of H-1PV interference with a factor determining the oncolytic activity of H-1PV. The ISG/HERV-shutdown thus provides a basis to generate a surrogate marker predicting the oncolytic efficacy of H-1PV in different patients.

## 4. Discussion

Our study demonstrates that in contrast to immune cells, tumor cells fail to recognize early parvoviral intermediates (e.g, replicative DNA forms mRF/dRF, dsRNA, and DNA:RNA). Passing this checkpoint is apparently required for a productive infection which is associated with an accidental global shutdown of the homeostatic innate immunity. Unexpectedly, non-structural parvoviral NS1/2 proteins interfere with the host machinery in a way which produces a global IFN/ISG-shutdown and the clade-specific disturbance of retrotransposons as a regulatory byproduct: associated with, but irrelevant for parvoviral replication in tumor cells. That collateral suppression of antiviral effectors occurs through or together with its major configurators, endogenous HERVs, although in cis modus cannot be excluded [42,64].

Bundling HERVs, IFNs, and ISGs to a homeostatic transcriptional module offers a novel possibility to explore host responses to parvoviral infection. First of all, the blunted interferogenicity of gemcitabine indicated at least a partial blockade along the cGAS/STING-TBK1 signaling pathway. The activation of cGAS/STING-signaling mediates immunogenic effects in dying tumor cells caused by radiotherapy, chemotherapy (including gemcitabine), adenoviral vectors, or oncolytic components [31,34,65,66,67,68]. cGAS/STING serves as cytoplasmic and ER sensor for diverse dsDNA, DNA:RNA hetero-duplexes, and viral Y-form DNA structures. cGAS recognizes duplex regions flanked by at least three unpaired guanosines at each end (G-YSD). Upon docking, this DNA ligand leads to the activation of cGAS and production of cyclic GMP-AMP, a second messenger which binds to STING and transduces the signal to TBK1 and IKKε kinases in order to phosphorylate interferon regulatory factors IRF3 and IRF7. Canonically, these transcriptional factors trigger the expression of IFN-I/III, which in turn activate ISRE (IFN-stimulated response element)-bearing ISG through the STAT1/STAT2/IRF9 complex [69,70,71,72].

Due to the known relations to protoparvoviruses, serine/threonine-protein kinase TBK1 [29], transcription factor IRF3 [73], and alarmin HMGB1 [9] proteins are the most promising candidates to control a homeostatic HERV/IFN/ISG module along the cGAS/STING-TBK1 pathway. The TBK1 deserves special attention, based on the report which describes an NS2-TBK1 interaction to prevent the latter from being recruited by STING [29]. This blockade impaired the antiviral response and antagonized the induction of IFN by Sendai virus in feline kidney fibroblast-like F81 cells infected with FPV. In addition to cGAS/STING, TBK1 may also transduce signals coming from TLR7 (viral ssRNA)/MyD88 and MAVS (viral dsRNA). Although the latter pathways are dispensable for an anti-parvoviral response [27], TBK1-blocking might increase the sensitivity of H-1PV-infected cells to other infections recognized by these pathways. IRF3 is of interest due to its ability to induce an IFN-independent ISG expression going in parallel with the susceptibility to atypical kinases known to be dysregulated by parvoviruses [73]. Our final candidate for cGAS/STING-inhibition is HMGB1, the only H-1PV-triggered ICD-determinant [9]. Recent reports endowed cGAS with an ability to sense long, HMGB1/TFAM-bound U-turn DNA by forming protein-DNA ladders. HMGB1 seems to pre-structure DNA to nucleate or stabilize cGAS dimers, with endogenous HMGB1 co-localizing with transfected DNA and cGAS in 85% of the observed cytosolic DNA loci. Most strikingly, high intracellular concentrations of HMGB1 abolished cGAS activity [71,74]. Further experiments are required to link the H-1PV-modified localization (nuclear/cytoplasmic), abundance, and redox status of host HMGB1 to the deactivation of cGAS/STING in infected tumor cells.

The sequestration of certain transcriptional factors might also yield a concomitant suppression of HERVs/IFNs/ISGs. In line with previous observations [45], our whole-genome screenings confirmed a strong suppression of NF-κB-regulated genes by H-1PV in PDAC cells. The sequestration of NF-κB by NS1 is known to weaken the expression of the exogenous retrovirus HIV-1 [38]. If H-1PV exerts such activity toward 5-LTR and 5-UTR-containing endogenous HERVs, it will result in the inhibition of autonomous retrotransposons and, consequently or in parallel, ISGs. Another candidate to consider is GC-binding factor SP1, occupying multiple binding sites in HERV/ISG and the parvoviral P4 promoter [75,76]. Binding/absorption of SP1 by overwhelming parvoviral DNA could sequester it from the host´s genomic DNA and thus reduce HERV/ISG-transcription.

Finally, a preliminary comparison of sensitive and resistant PDAC cell lines revealed “E2F targets” as the top enriched GSEA-signature (NES = +3.0; *p* > 0.0001). Instrumental for parvoviral replication, E2F also regulates DNA methyltransferase 1 (DNMT1). Epigenetic modifier is a key factor in the regulation of HERVs and ISGs. Goel et al. found that the suppression of the E2F-target DNMT1 triggers the expression of HERVs, IFN-III, and ISGs under CDK4/6-inhibitory drugs promoting anti-tumor immunity in colon cancer cells [77]. In PDAC cell lines, basal DNTM1 levels inversely correlated with the IC50 doses (R = −0.70; *p* = 0.036); H-1PV elevated DNMT1 expression, in resistant cells less than in sensitive cells (Figure S6). Whether DNMT1 is indeed a major byproduct of H-1PVs interference with E2F to control a HERV/IFN/ISG-module and a suitable surrogate marker to predict therapy’s efficacy requires experimental validation.

The ability of H-1PV to reduce homeostatic ISGs in malignant cells might be a unique feature to be exploited by parvovirus-based therapies. It should be studied in more detail, especially as the intrinsic interferon program highlights cancer stem cells resisting many other infections [78,79]. The reduction of HERV and ISG expression in PDAC cells reduces their malignant potential and improves the sensitivity to chemo-/radiotherapy and virally mediated gene therapy [61,80,81]. Concerning immunogenic cell death, H-1PV does not trigger the release of IFN-I in tumor cells to overcome ecto-CALR failure. Moreover, it abolishes the action of supplementary interferogenic drugs like gemcitabine, a standard chemotherapeutic used to treat PDAC. At first sight, this behavior casts doubt on the feasibility of ICD completion employing parvovirus-based protocols. However, IFN expression was clearly detectable in infected human PBMCs and in circulation of infected mice [82]. Considering the latter as an in vivo proof of robust production of IFN by immune cells, one can suggest that these will provide type-I IFN upon intra-tumoral injection of H-1PV. The insensitivity of H-1PV expression to IFN in tumor cells and the notorious cellular heterogeneity of PDAC tumors thus become an advantage [83]. Frequently occupying less than a quarter of PDAC lesions, malignant cells are embedded in a stromal microenvironment full of non-transformed cells: myofibroblasts, nerves and vessels, as well as leukocytes. Immune and stromal signatures bear a prognostic relevance [22,23,24,25]. In this context, not only direct oncolytic activity but also the infiltration of cancerous lesions with immune cells and their close proximity to dying tumor cells matter for ICD efficacy and the therapeutic outcome. These observations imply that the combined treatment will allow for intra-tumoral accumulation of IFN by reassigning its source from “blocked” infected PDAC cells to “activated” infected immune cells. Finally, the global suppression of ISGs in the tumor cell might improve chemo- and radio-sensitivity in tumor cells. Thus, H-1PV should be tested for synergisms with drugs and other oncolytic viruses for which the efficacy is impaired by high ISG levels.

## 5. Conclusions

The oncolytic H-1PV causes a global deferred collapse of homeostatic innate immunity through and/or together with the suppression of HERVs, a previously unknown event occurring in tumor cells. Most likely, the ISG-shutdown is irrelevant for parvoviral replication but it could be used as a basis to develop a surrogate marker for the oncolytic efficacy of H-1PV among patients. Furthermore, H-1PV may impair the interferogenicity while boosting the cytotoxicity of co-applied drugs.

## Data Availability

The RNAseq data are available under the GEO Accession number GSE160322 and GSE160434.

## Supplementary Materials

The following are available online at https://www.mdpi.com/article/10.3390/v13061019/s1, Supplementary Figures S1–S6.
