# Supplementary material for: Human Retrotransposons and the Global Shutdown of Homeostatic Innate Immunity by Oncolytic Parvovirus H-1PV in Pancreatic Cancer
Source: Viruses. 2021 May 28;13(6):1019. doi: 10.3390/v13061019 (PMC8228339; doi:10.3390/v13061019)
Supplement: Supplementary file 1 [file viruses-13-01019-s001.zip › viruses-1206357-supplementary.pdf]

viruses

MDPI

Supplementary

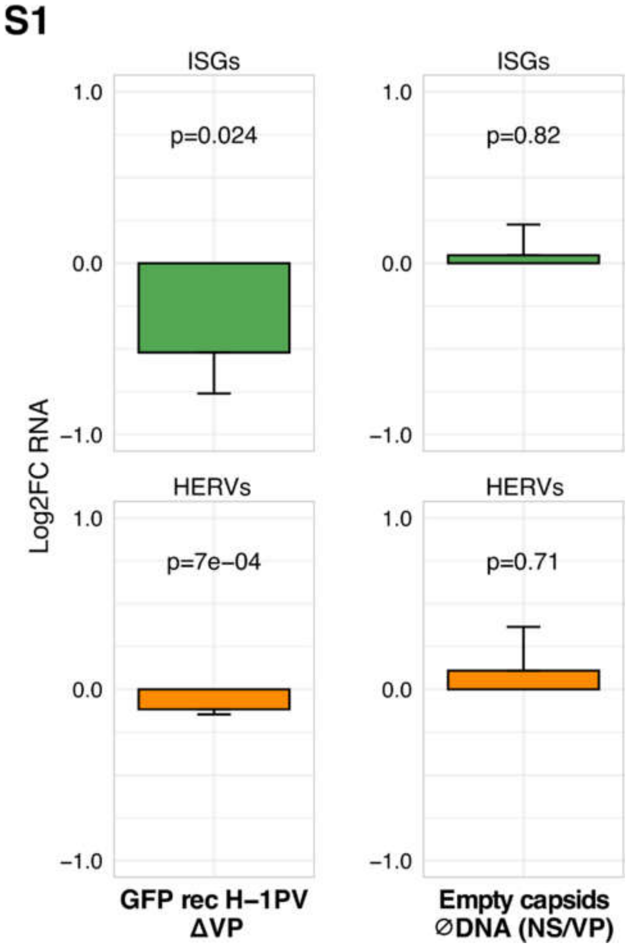

Figure S1. Non-structural NS1/NS2 proteins but not capsid VP1/2 proteins are essential for ISG suppression by H-1PV in pancreatic tumor cells. The graph summarizes the data of the experiments with three PDAC cell lines (AsPC1, MiaPaca2, and T3M4), which were infected for 72 hours with the viral particles constructed to lack the genes encoding only nonstructural proteins NS1/NS2 (recombinant virus H-1PV-GFP) or both, NS1/NS2 and capsid VP1/2 proteins (empty capsids). The expression of three markers ISGs (ISG15, IFITM1 and OAS1) and three HERVs (HERV-Kenv, HERV-Wpol, HERV-W1env) was measured by qRT-PCR and processed to log2FC+SEM values as described in Material and Methods, analogous to shown in Figure 1.

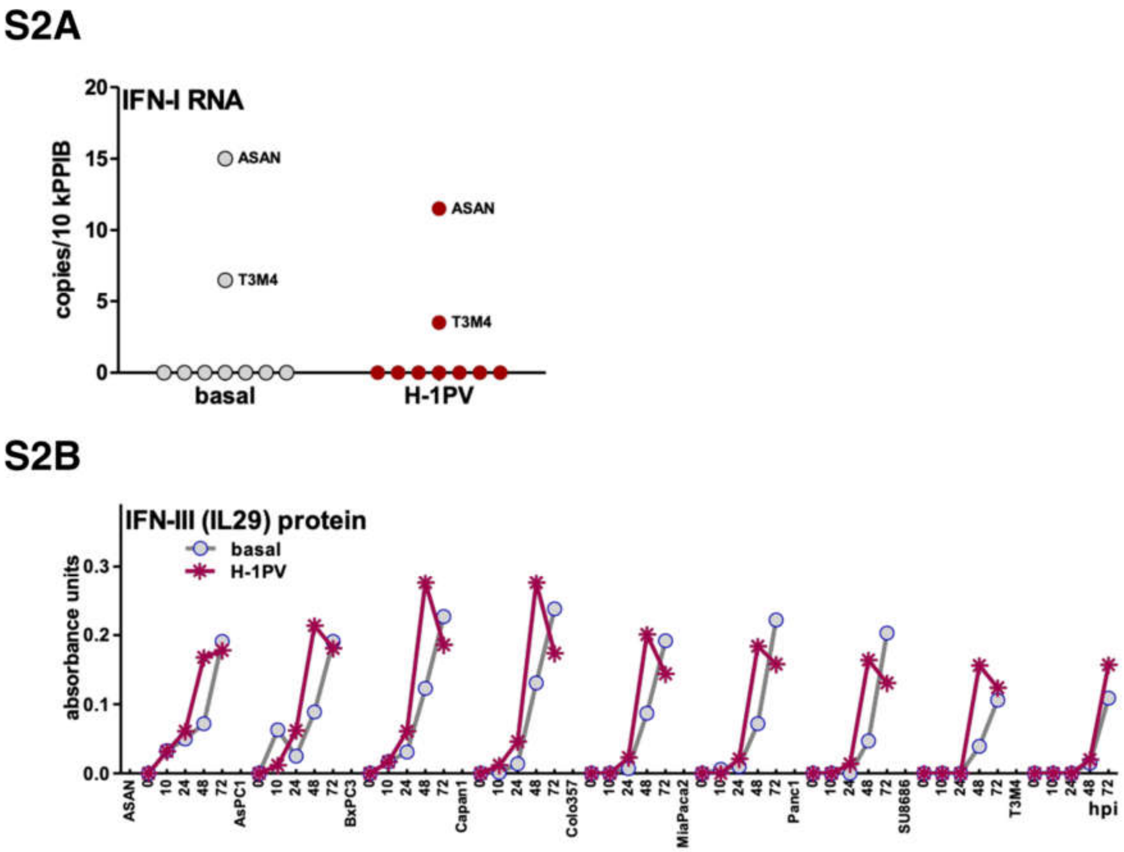

Figure S2. Impact of H-1PV on IFN-I and IFN-III in pancreatic tumor cells. (a) Expression of type I IFN (IFNα and IFNβ) was measured in H-1PV-infected PDAC cells (n=9) by qRT-PCR. Shown are mean values for both subtypes at 72 hpi. **(b)** Accumulation of the IFN-III (IL29) was measured by a commercial ELISA kit (Invitrogen Thermo Scientific) in the supernatants of the PDAC cell lines collected from 0 to 72 hours of the culture with or without H-1PV infection. The accumulated protein levels are shown as the absorbance A_{450nm} units blanked against values measured for the culture medium alone.

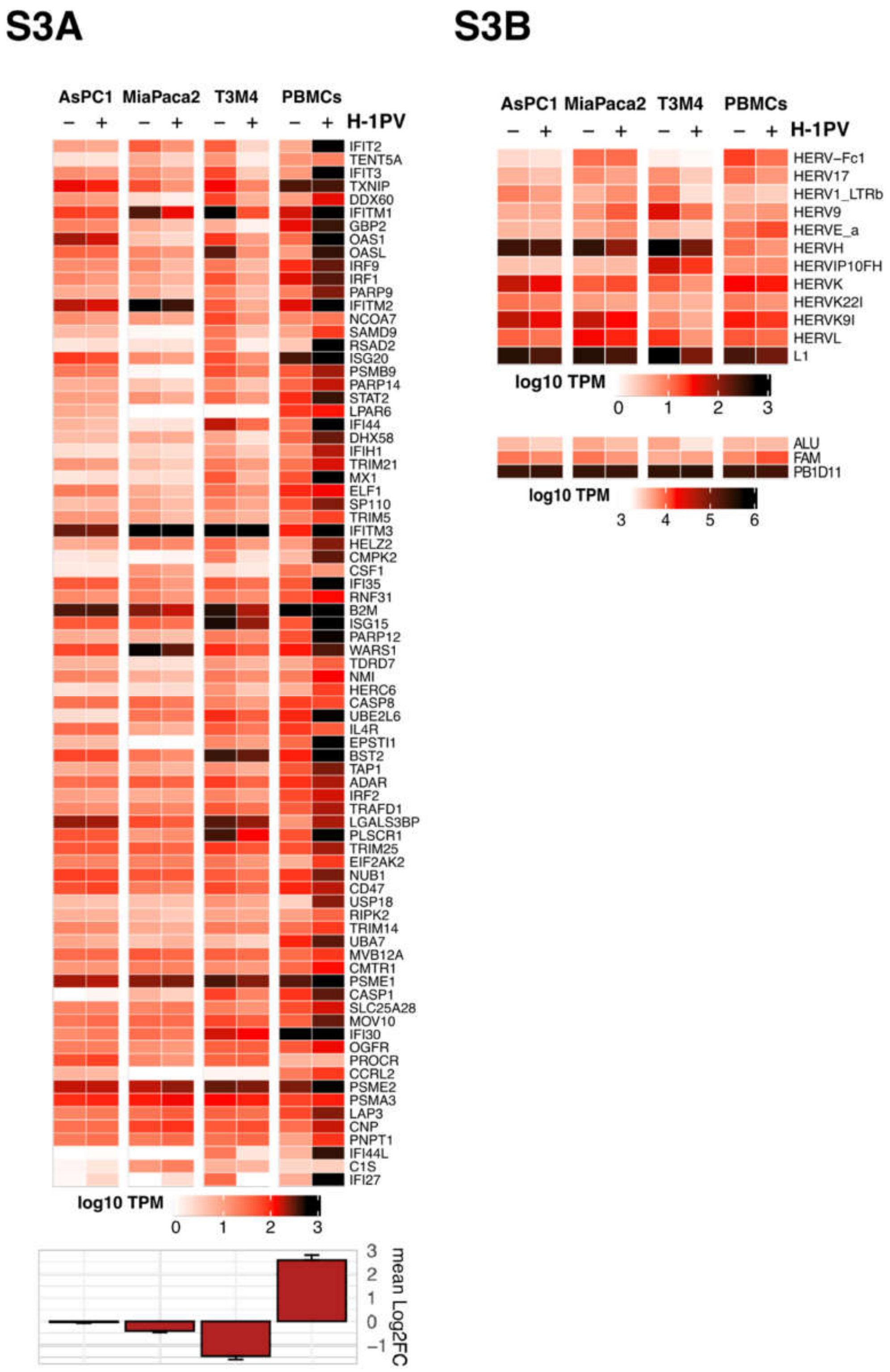

Figure S3. Global shutdown of innate immunity and HERVs in tumor cells. (a) Heatmap showing absolute RNA expression of the IFN-alpha response genes as log10-transformed TPM values to illustrate differences across treatment groups, cells and particular transcripts. The graph below summarizes the presented findings above in form of Log2FC±SEM. **(b)** Expression of the most representative/abundant repetitive elements presented as heatmap analogous to Panel A.

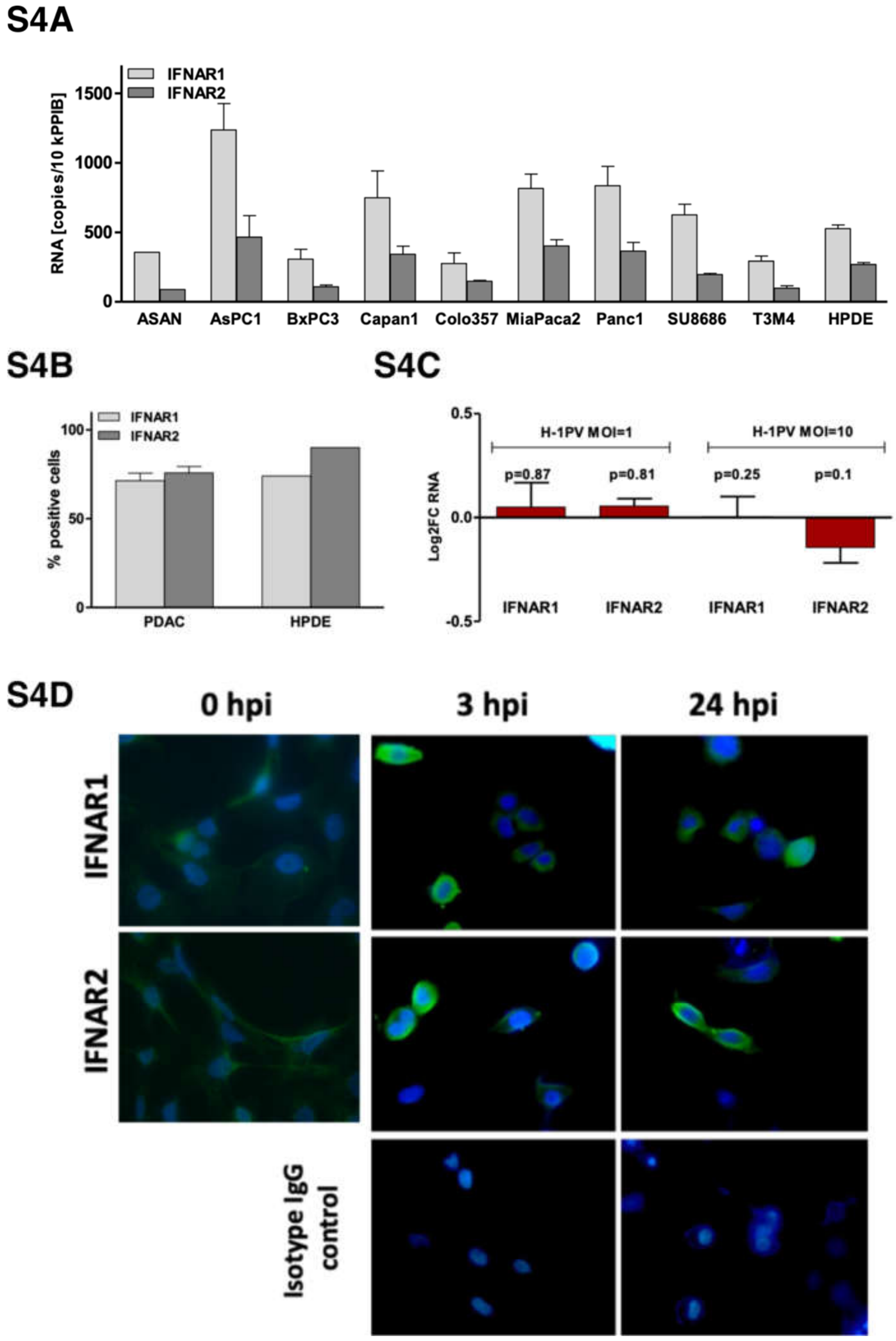

Figure S4. Pancreatic tumor cells are well equipped with IFN-I receptors IFNAR1 and IFNR2. (a) RNA expression of IFNAR1 and IFNAR2 was measured by qRT-PCR in nine pancreatic tumor cell cells and compared to that in the immortalized normal pancreatic ductal epithelial cells (HPDE, a gift from Dr. Ming-Sound Tsao, FRCPC, University Health Network, Canada). **(b)** Pancreatic tumor cell lines and HPDE were stained with the mouse antibodies against human IFNAR1 and IFNAR2 (PBL Interferon Source, Piscataway, NJ, USA) using standard immunofluorescence method. For controls, we replaced primary antibodies with respective isotype controls, IgG1 or IgG2a. Followed exposure to the secondary anti-mouse IgG antibodies labeled with fluorescent marker Cy2 (green) and to the nuclear stain DAPI (blue), Cy2-positive and negative cells were manually counted using fluorescence Zeiss Axioplan2 microscope (Oberkochen, Germany) and related to each other to calculate % of IFNAR1 and IFNAR2-positive cells. The staining pattern was further confirmed by FACS. **(c)** Upon infection of the pancreatic tumor cells with H-1PV at MOI=1 and MOI=10 PFU/cell for 24 hours, IFNAR1 and IFNAR2 RNA expression was measured by qRT-PCR in control and infected cultures and transformed to show log2FC.

(d) An illustrative immunofluorescent image showing IFNAR1- and IFNAR2-positive cells in H-1PV-infected T3M4 cultures.

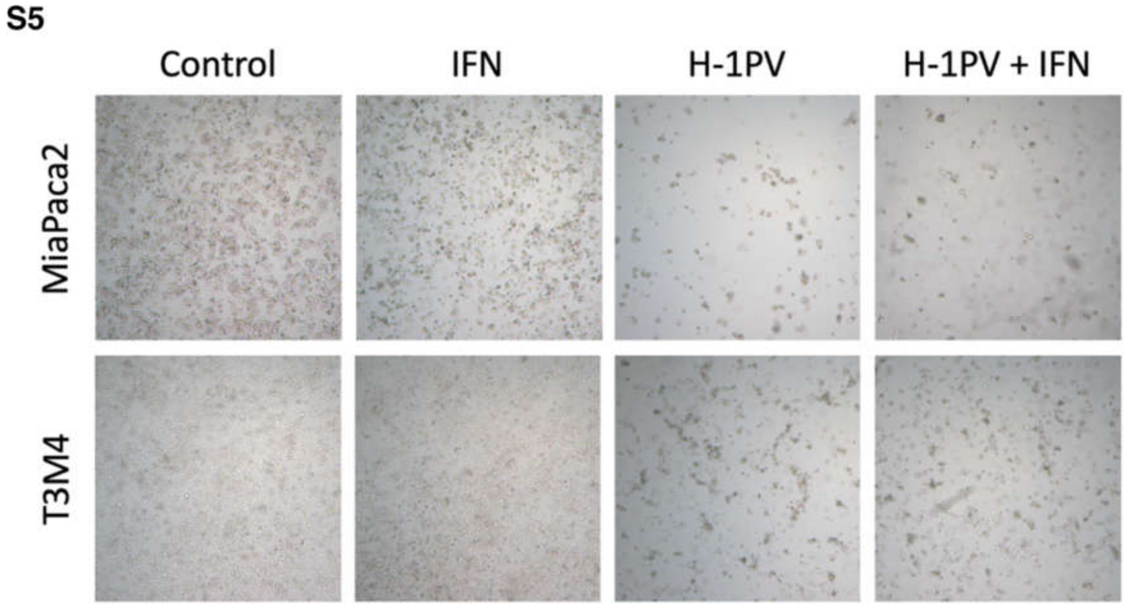

Figure S5. H-1PV is resistant to IFN-I. Retention of the oncolytic power of H-1PV in IFNα-treated PDAC cells as illustrated by their morphological appearance after 72 hours of combined exposure.

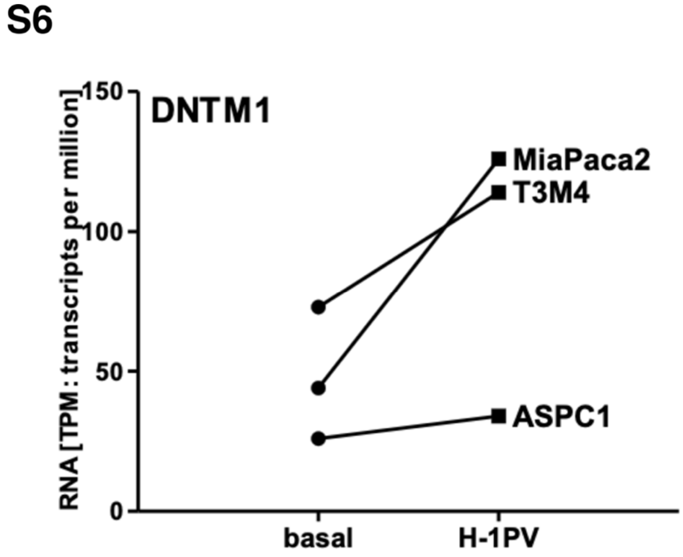

Figure S6. Upregulation of E2F-target, DNA methyltransferase 1 (DNMT1) by H-1PV in pancreatic tumor cells. The RNAseq data show RNA expression level of DNTM1 in three infected PDAC cells at 48 hours post infection.
